# Integrating Ophthalmology, Endocrinology, and Digital Health: A Bibliometric Analysis of Telemedicine for Diabetic Retinopathy

**DOI:** 10.3390/healthcare14020183

**Published:** 2026-01-12

**Authors:** Theofilos Kanavos, Effrosyni Birbas

**Affiliations:** 1 Northwell Health, New Hyde Park, NY 11042, USA; ebirbas@northwell.edu; 2 Institute of Health System Science, Feinstein Institutes for Medical Research, Northwell Health, Manhasset, NY 11030, USA; 3 Institute of Bioelectronic Medicine, Feinstein Institutes for Medical Research, Northwell Health, Manhasset, NY 11030, USA; 4 Elmezzi Graduate School of Molecular Medicine, Northwell Health, Manhasset, NY 11030, USA

**Keywords:** telemedicine, telehealth, telecare, teleophthalmology, diabetes mellitus, diabetic retinopathy, screening, monitoring, fundus photography, artificial intelligence

## Abstract

**Background/Objectives:** Telemedicine has emerged as a pivotal approach to improving access to diabetic retinopathy (DR) screening, diagnosis, management, and monitoring. Over the past two decades, rapid advancements in digital imaging, mobile health technologies, and artificial intelligence have substantially expanded the role of teleophthalmology in DR, resulting in a large volume of pertinent publications. This study aimed to provide a scientific overview of telemedicine applied to DR through bibliometric analysis. **Methods:** A search of the Web of Science Core Collection was conducted on 15 November 2025 to identify English-language original research and review articles regarding telemedicine for DR. Bibliographic data from relevant publications were extracted and underwent quantitative analysis and visualization using the tools Bibliometrix and VOSviewer. **Results:** A total of 515 articles published between 1998 and 2025 were included in our analysis. During this period, the research field of telemedicine for DR exhibited an annual growth rate of 13.14%, with publication activity markedly increasing after 2010 and peaking in 2020–2021. Based on the number of publications, United States, China, and Australia were the most productive countries, while *Telemedicine and e-Health*, *Journal of Telemedicine and Telecare*, and *British Journal of Ophthalmology* were the most relevant journals in the field. Keyword co-occurrence analysis revealed three major thematic clusters within the broader topic of telemedicine and DR, namely, public health-oriented work, telehealth service models, and applications of artificial intelligence technologies. **Conclusions:** The role of telemedicine in DR detection and care represents an expanding multidisciplinary field of research supported by contributions from multiple authors and institutions worldwide. As technological capabilities continue to evolve, ongoing innovation and cross-domain collaboration could further advance the applications of teleophthalmology for DR, promoting more accessible, efficient, and equitable identification and management of this condition.

## 1. Introduction

Diabetes mellitus (DM) represents a complex metabolic disorder characterized by chronic hyperglycemia due to impaired insulin secretion or action. Diabetic retinopathy (DR) constitutes a sight-threatening microvascular complication of uncontrolled DM affecting the retinal blood vessels [1]. This condition, which impacts around one-third of patients with DM [2], is a leading contributor to vision loss globally and the most common cause of preventable blindness among working-age adults [3]. The global increase in DM prevalence is accompanied by a corresponding rise in its complications, including DR. Importantly, visual impairment resulting from DR adversely affects patients’ quality of life and compromises their ability to effectively manage their condition [4]. In addition, DR imposes a major public health concern due to the considerable economic burden associated with its visual consequences, encompassing both direct expenditures of medical interventions and indirect costs related to reduced workforce productivity [5]. Although early detection and timely treatment of DR can lower the risk of severe vision loss by about 90%, the adherence of patients with DM to DR screening remains suboptimal. This pitfall is largely attributed to the frequently high costs and limited accessibility of eye care services [6].

Telemedicine, broadly defined as the delivery of healthcare services—including screening, diagnosis, treatment, and monitoring—over distance using digital communication technologies [7], offers a promising approach to the care of patients at risk of or living with DR. Teleophthalmology in the context of DR represents an innovative strategy for retinal evaluation, helping preserve vision and alleviate the overall burden on healthcare systems. It is considered a reliable and cost-effective complement to traditional care models because of its capacity to reduce barriers and enhance accessibility to specialized eye care across both urban and rural settings [8]. In recent years, the advent of ultra-widefield fundus photography [9], the rise of smartphone-based techniques capable of capturing high-quality ocular images [10], and the explosive growth of artificial intelligence (AI)-driven image analysis [11] have substantially advanced the applicability of teleophthalmology for a range of ocular conditions, including DR.

Although previous bibliometric analyses have examined topics such as AI in telemedicine [12], global trends in DR research [13], and AI in DR [14], a dedicated article to comprehensively capture the structure, evolution, and thematic focus of research on teleophthalmology for DR has been lacking. To address this gap, the aim of the present study was to conduct a targeted bibliometric analysis to systematically map and quantitatively describe the current scholarly landscape of telemedicine for DR.

## 2. Materials and Methods

This study adopted a bibliometric research design and followed established methodological guidelines for conducting bibliometric analyses, as described by Passas [15], Donthu et al. [16], and Gan et al. [17]. In accordance with these recommendations, we employed a transparent and reproducible workflow consisting of defining the research objectives, collecting data from relevant literature, analyzing and visualizing the data, and finally interpreting and reporting the findings.

### 2.1. Data Source

The Web of Science Core Collection was selected as our source of data for this bibliometric analysis, as it constitutes a comprehensive and multidisciplinary bibliographic database that provides curated coverage of high-quality literature across the medical and technological fields. Its key asset for bibliometric research lies in the depth and uniformity of the bibliographic information it provides, including publication title, abstract, authors, affiliations, keywords, document type, references, and citations [18,19]. To ensure maximal coverage of the literature, we included all component indexes of the Web of Science Core Collection, encompassing the Science Citation Index Expanded, Social Sciences Citation Index, Arts & Humanities Citation Index, Emerging Sources Citation Index, Book Citation Index, Conference Proceedings Citation Index, Index Chemicus, and Current Chemical Reactions.

### 2.2. Search Strategy and Data Collection

A comprehensive search query was constructed to identify publications on the applications of telemedicine in DR. To ensure acceptable precision while maintaining high sensitivity, we searched the title, author keywords, and KeyWords Plus fields using a combination of terms related to telemedicine and DR. The exact search query we used was the following: (TI = (telemedicine OR tele-medicine OR telehealth OR tele-health OR teleophthalmology OR tele-ophthalmology OR “digital health” OR eHealth OR “mobile health” OR mHealth OR “remote monitoring” OR “remote screening” OR “remote management”) OR AK = (telemedicine OR tele-medicine OR telehealth OR tele-health OR teleophthalmology OR tele-ophthalmology OR “digital health” OR eHealth OR “mobile health” OR mHealth OR “remote monitoring” OR “remote screening” OR “remote management”) OR KP = (telemedicine OR tele-medicine OR telehealth OR tele-health OR teleophthalmology OR tele-ophthalmology OR “digital health” OR eHealth OR “mobile health” OR mHealth OR “remote monitoring” OR “remote screening” OR “remote management”)) AND (TI = (“diabetic retinopathy” OR “diabetic macular edema”) OR AK = (“diabetic retinopathy” OR “diabetic macular edema”) OR KP = (“diabetic retinopathy” OR “diabetic macular edema”)). By using filters, our search was limited to original research articles and reviews written in English. No time restrictions were applied because the publications captured by the above search strategy naturally spanned only from 1998 to 2025, a timeframe not sufficiently long to warrant temporal truncation. The database search was conducted on 15 November 2025.

All retrieved records were exported as plain text files in “Full Record and Cited References” format to ensure compatibility with the bibliometric software tools we used in our analysis. Subsequently, the dataset underwent a two-step screening process conducted in Microsoft Excel. First, duplicate entries were identified and removed. Second, titles and abstracts were manually screened to assess eligibility. To be included in our study, articles needed to be pertinent to both telemedicine and DR. Documents deemed irrelevant to either of these domains were excluded. The remaining publications constituted the final dataset for our bibliometric analysis.

### 2.3. Data Analysis and Visualization

Bibliometric analysis is a systematic method for quantitatively evaluating scientific literature, facilitating the identification of patterns, trends, and impact within a field [15]. By exploring and analyzing large volumes of scientific data in a rigorous way, it enables researchers to map the intellectual structure of a certain domain, track its evolution over time, and highlight emerging topics [16]. In the context of telemedicine for DR in particular, such analysis of scholarly literature offers a global perspective on the distribution of research activity and dominant thematic areas. To conduct our bibliometric analysis, we utilized two widely used software tools in scientometrics, namely, Bibliometrix version 5.2.0 [20] and VOSviewer version 1.6.20 [21].

Bibliometrix is an open-source R package that offers all the main bibliometric methods of analysis and is extensively used for quantitative research in bibliometrics and scientometrics. For this study, we employed Biblioshiny, the graphical web-based interface of Bibliometrix, which facilitates interactive data processing and visualization without requiring direct R scripting [20]. Specifically, Biblioshiny was used to (1) analyze the annual number of publications and citations, (2) calculate the annual growth rate of publications and characterize the expansion of research activity over time, (3) identify the most relevant authors, journals, institutions, and countries, (4) determine the most cited documents, references, authors, journals, institutions, and countries, (5) evaluate the impact of authors and journals, (6) assess the productivity of authors, journals, institutions, and countries, and (7) examine the most frequently occurring keywords. For all keyword-based analyses, a predefined synonym list was implemented within Biblioshiny to merge variably spelled or semantically equivalent terms, ensuring that conceptually identical keywords were treated as a single item. Additionally, for analyses related to authors, name variants, such as differences in abbreviations and punctuation, were manually harmonized so that all publications corresponding to the same individual were consistently attributed to a single standardized name.

VOSviewer is a freely available program designed for constructing and visualizing bibliometric networks. Its strength lies in its ability to generate intuitive graphical maps that depict relationships among items such as keywords or countries [21]. In our study, this software was used to perform and visualize the results of (1) keyword co-occurrence and (2) country-level co-authorship analyses. To ensure consistency and interpretability of the keyword co-occurrence maps, a customized thesaurus file was applied to merge different variants of the same keyword and exclude overly broad or irrelevant terms that did not contribute substantively to the thematic structure of the field.

## 3. Results

### 3.1. Dataset Characteristics

**Figure 1:**
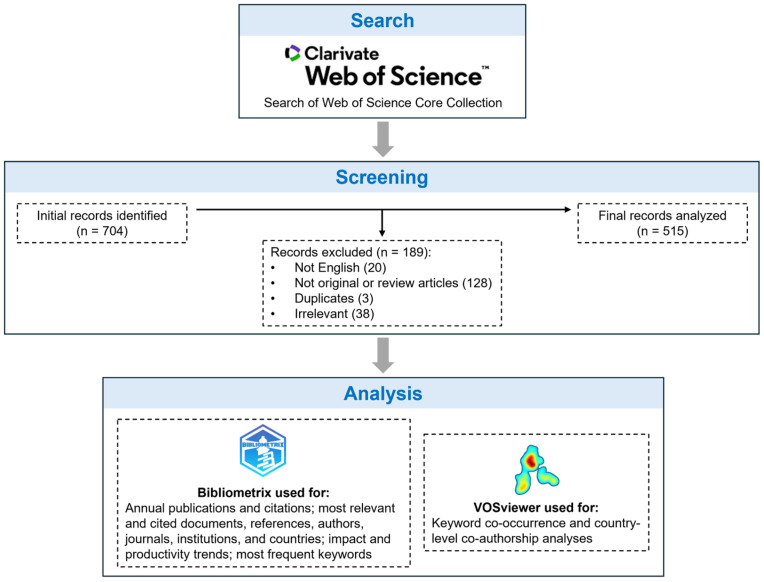
Overview of the study workflow, including data retrieval, screening of records, and subsequent analysis using specialized software.

The entire process of record identification, screening, and final analysis is summarized in Figure 1. Our systematic search initially yielded 704 records, 189 of which were excluded through filtering and screening. Consequently, 515 unique original research or review articles written in English and relevant to both telemedicine and DR were included in our analysis.

These 515 documents were published across 154 different journals and were authored by 2810 individual investigators, with only 12 papers written by a single author. The average number of co-authors per article was 6.7. Nearly one-quarter of all publications involved international collaboration with co-authors from more than one country. The average age of documents was 7.38 years, while the included publications accrued a mean of 35.19 citations each.

### 3.2. Publication Output

**Figure 2:**
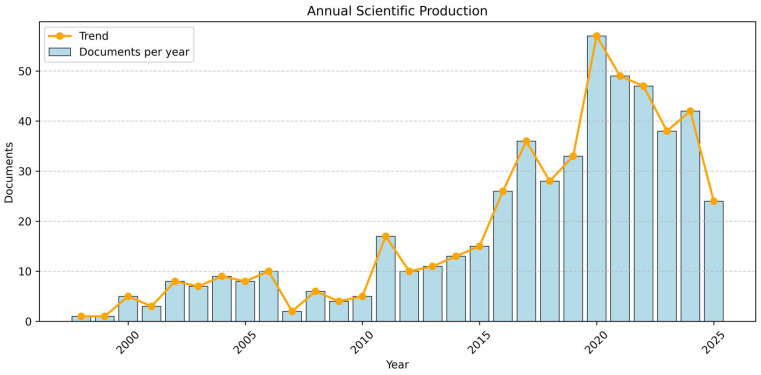
Annual scientific production showing the number of studies published per year in the field of telemedicine and diabetic retinopathy. The visualization was generated in Python version 3.12.12.

Annual scientific production in the field of telemedicine and DR has generally increased over the study period although the growth has been uneven. Publication activity remained relatively modest until 2010 but rose rapidly thereafter, reaching a peak of 57 articles in 2020 and 49 in 2021 (Figure 2). This surge is unsurprising given that the onset of coronavirus disease 2019 (COVID-19) pandemic in 2020 substantially accelerated the adoption and evaluation of remote healthcare models, including teleophthalmology [22]. Despite the fluctuations observed, the overall trajectory demonstrated a clear expansion of research activity, reflected by an annual growth rate of 13.14%.

**Figure 3:**
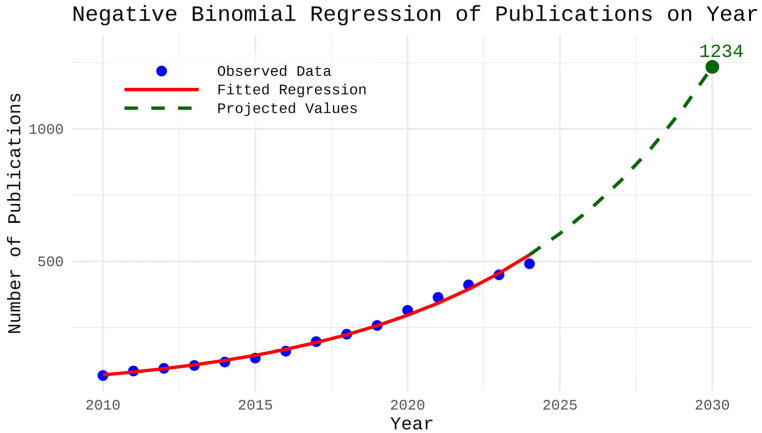
Negative binomial regression of cumulative publication count in the field of telemedicine and diabetic retinopathy from 2010 to 2024, with projected values through 2030. Analysis was performed in R version 4.5.2.

To further characterize the growth trajectory of the field, we conducted a predictive analysis using generalized linear regression. We restricted our dataset for this purpose to the period 2010–2024 because 2010 marked the beginning of meaningful and consistent publication activity, while 2024 was the most recent complete year. To avoid bias, publications from 2025 were excluded from this analysis because that year was ongoing at the time of data collection and therefore not fully documented. Examination of cumulative publication counts up to each year revealed substantial overdispersion, with variance far exceeding the mean, indicating that a negative binomial regression model was more appropriate than a Poisson model for describing the relationship between total publication count and year. Using this model, publication activity was projected through 2030. The analysis demonstrated a continued upward trajectory, suggesting that research on telemedicine applications for DR is likely to remain an active area of scientific interest over the upcoming years, with the expected number of publications exceeding 1000 by 2030 (Figure 3).

### 3.3. Country Analysis

**Figure 4:**
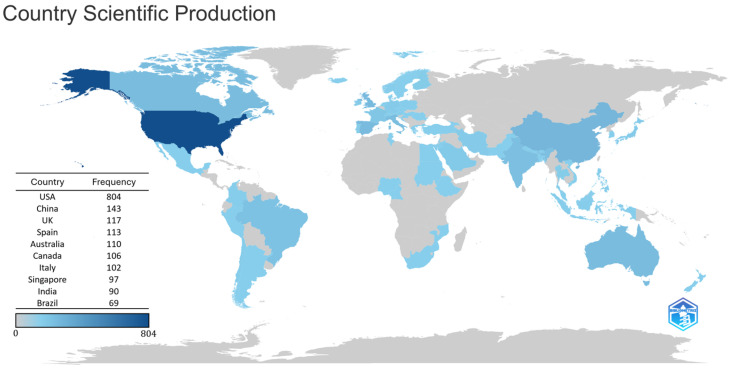
World map depicting country-level scientific production in the field of telemedicine and diabetic retinopathy, measured by the number of times each country appeared in the affiliation lists of pertinent publications. Abbreviations: UK, United Kingdom; USA, United States of America.

**Figure 5:**
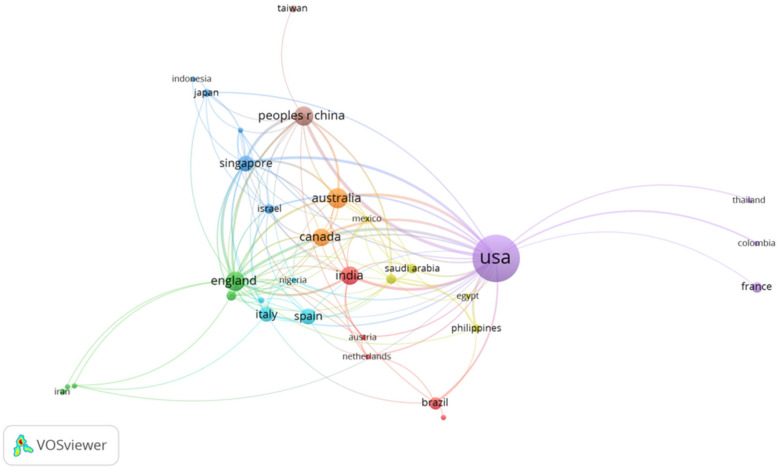
International country-level collaboration network based on co-authorship in the field of telemedicine and diabetic retinopathy. Node size reflects the publication output of each country, while link thickness indicates the strength of cooperation between countries.

Country-level scientific production showed substantial global engagement in telemedicine and DR research, with contributions from a total of 61 countries. Based on the corresponding author’s institutional affiliation, the United States produced the largest number of publications, followed by China, Australia, Canada, Spain, India, Singapore, the United Kingdom, Italy, and Brazil (Table 1). Among these leading contributing countries, Singapore exhibited the highest number of average citations per article as well as the largest proportion of multiple-country publications. A world map of scientific production illustrating the global distribution of research activity by depicting how frequently each country appeared in the affiliation lists of the included publications is presented in Figure 4. In addition, a country-level co-authorship network visualizing international research cooperation patterns and the strength of collaborative ties between countries is shown in Figure 5.

### 3.4. Journal Analysis

**Figure 6:**
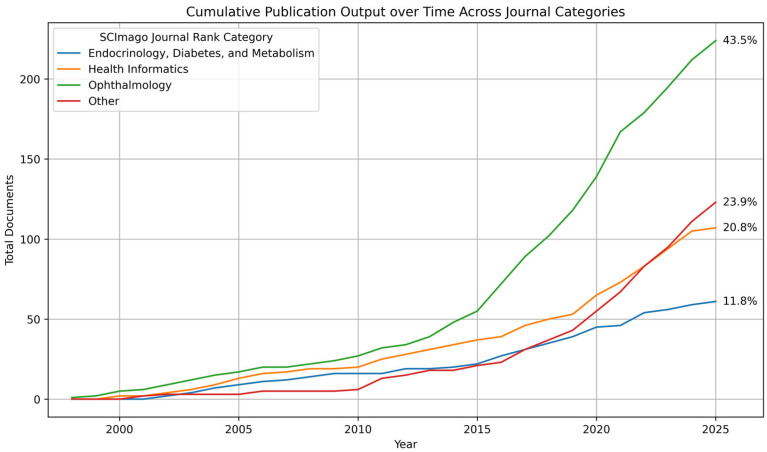
Publication output over time in the field of telemedicine and diabetic retinopathy, stratified by journal category according to the SCImago Journal Rank. The visualization was generated in Python version 3.12.12.

A total of 154 journals have published scientific research related to telemedicine and DR. When ranked by the number of publications in the field, *Telemedicine and e-Health* emerged as the most productive source, followed by the *Journal of Telemedicine and Telecare*, both classified under the Health Informatics category in the SCImago Journal Rank. Among the remaining eight journals in the list of the 10 sources with the highest publication counts, seven were ophthalmology-focused and one was endocrinology-oriented. This distribution underscores the multidisciplinary nature of the field, which relies on collaboration among ophthalmologists, endocrinologists, and health informaticians. The positioning of this research field at the intersection of ophthalmology, endocrinology, and health informatics is further highlighted in Figure 6. Details on the local productivity and impact of the journals with the highest publication counts are shown in Table 2.

### 3.5. Author and Affiliation Analysis

Articles related to telemedicine and DR were authored by a total of 2810 individual investigators. Local productivity and impact details for the authors with the greatest number of publications in the field are presented in Table 3.

In terms of institutions, a wide international network supported scientific output in the field of telemedicine and DR. Major centers of research activity included University of Harvard, the University of California System, the University of London, the Veterans Health Administration, the National University of Singapore, the University of Michigan, Johns Hopkins University, the University of Alabama, the University of Toronto, and the Singapore National Eye Center. Notably, numerous other institutions worldwide also played critical roles in advancing this research area.

### 3.6. Document Analysis

The most impactful publications in the field based on total citation count and stratified by document type are presented in Table 4. Because original research articles and reviews differ fundamentally in their objectives, methodological approaches, and contributions to the literature, a decision was made to analyze these two document types separately. The 10 most cited original articles were published across eight different journals, with *IEEE Transactions on Medical Imaging* and *Ophthalmology* each appearing twice in the list. Meanwhile, the 10 most cited review articles were published in eight different journals, with the *British Journal of Ophthalmology* and *Telemedicine and e-Health* each represented twice. With respect to the content of these 10 original articles, five were directly relevant to the applications of AI in ophthalmology [25,26,27,28,29], reflecting the central role of computer-aided image analysis, segmentation, and classification in advancing the field. Similarly, another article developed an algorithm for the automated segmentation of optic disc in digital fundus photographs [30]. The remaining articles focused on the evaluation of the clinical and economic effectiveness of telemedicine-based programs for DR screening across diverse healthcare settings [31,32,33], as well as on clinical recommendations for the comprehensive management of DM [34]. A similar thematic pattern was observed among the most cited review articles, with four of the 10 publications centered on the role of AI in DR and ophthalmology in general [35,36,37,38].

### 3.7. Keyword Frequency and Co-Occurrence

**Figure 7:**
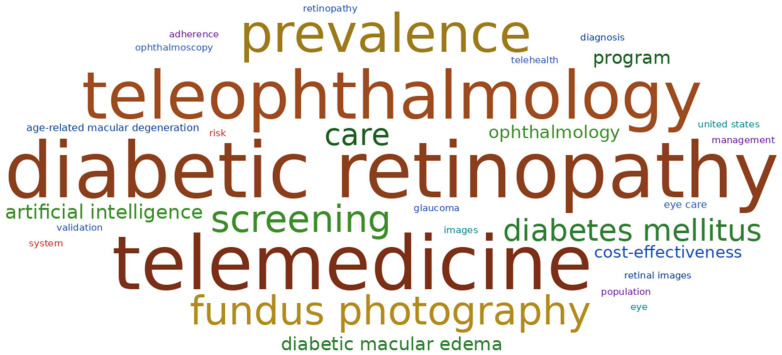
Word cloud showing the most frequently occurring keywords in the field of telemedicine and diabetic retinopathy. The visualization was generated in Python version 3.12.12.

The analysis of keyword frequency revealed a concentrated set of terms that reflect the major thematic directions of research in telemedicine for DR. Apart from generic terms like ophthalmology, telemedicine, diabetic retinopathy, and diabetes mellitus, several other keywords appeared repeatedly, including artificial intelligence and related concepts, fundus photography, camera, images, diagnosis, screening, prevalence, population, risk factors, and cost-effectiveness. Together, these terms highlight the field’s strong interest in remote image-based technologies, potentially enhanced by the power of AI, for diagnostic, screening, and epidemiological purposes. A visual representation of keyword frequency is provided in Figure 7, illustrating the prominence of the most recurrent concepts within the field.

To further examine the role of ocular imaging technologies in the research field of telemedicine for DR, we performed an exploratory keyword-based assessment focusing on imaging-driven and smartphone-enabled approaches. Ocular imaging-related terms were present as keywords in 46.80% of the analyzed studies, accounting for 7.61% of all keyword occurrences within the bibliometric dataset. By contrast, smartphone-related terms appeared as keywords in 6.21% of documents, representing 0.75% of all keyword instances. Collectively, these findings quantitatively indicate a strong emphasis of this research field on imaging-oriented strategies while highlighting the emergence of smartphone-centered work as a distinct subset within this broader body of literature.

**Figure 8:**
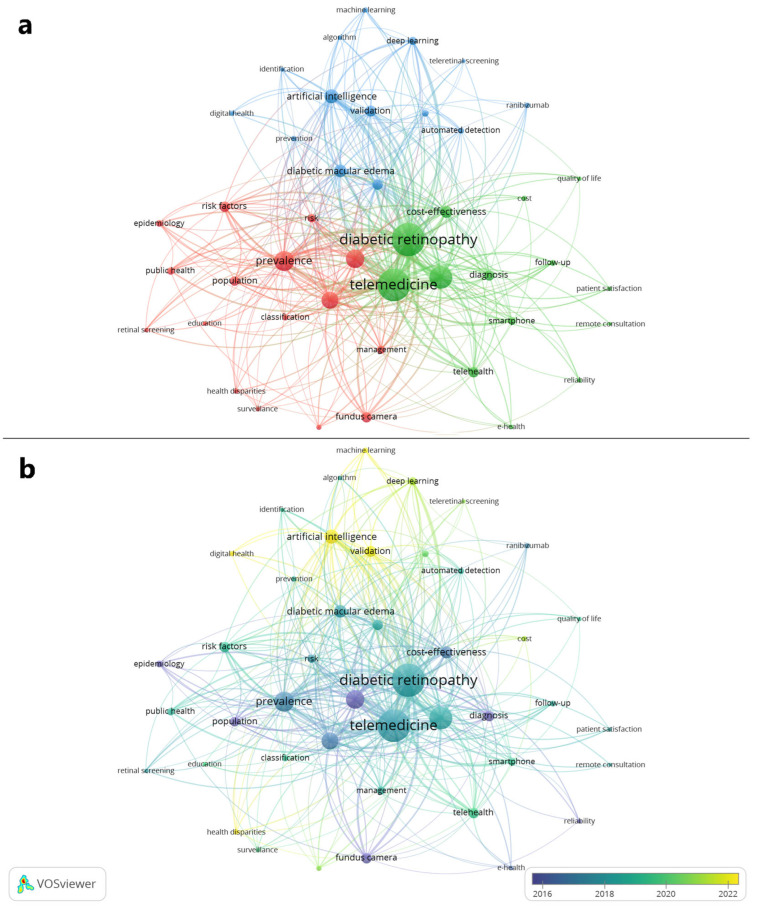
Keyword co-occurrence mapping of research on telemedicine for diabetic retinopathy. (**a**) Network visualization showing three thematic clusters representing public health-related applications in red, telehealth service models in green, and machine learning-driven technologies in blue; (**b**) overlay visualization illustrating the emergence of recent research trends with emphasis on artificial intelligence and health disparities.

The keyword co-occurrence analysis identified three major thematic clusters within the broader field of telemedicine for DR, as illustrated in Figure 8a. The first cluster, colored red in the network visualization, centered on population-based and public health-oriented work related to epidemiology, prevalence, risk factors, screening, surveillance, and health disparities. The second cluster, shown in green, focused on healthcare delivery and clinical service models based on telemedicine as well as the patient satisfaction and cost-effectiveness associated with them. The third cluster, depicted in blue, encompassed topics related to machine learning-driven technologies to facilitate telecare in DR. Together, these clusters highlight how the field integrates public health needs, telehealth service models, and advanced computational methods to support remote detection, management, and follow-up of DR. Furthermore, the overlay visualization in Figure 8b highlighted the growing interest in machine learning, and particularly deep learning, applications for assisting the approach to DR in the realm of teleophthalmology.

## 4. Discussion

This bibliometric analysis showed a rapidly expanding body of literature at the intersection of telemedicine and DR, consistent with prior narrative and systematics reviews [44,45]. The publication growth in this field accelerated markedly after 2010 and peaked during the COVID-19 era. This trajectory aligns with broader trends in telemedicine. Specifically, telehealth visits increased by 154% in the last week of March 2020 compared to the same period of the previous year, reflecting the multiple benefits of telemedicine during a pandemic, such as expansion of access to care, reduction in disease exposure for patients and providers, and preservation of scarce personal protective supplies [46].

The keyword co-occurrence analysis identified three major thematic clusters within the field of DR and telemedicine. The first cluster focused on DR as a public health concern, highlighting its status as the leading cause of vision loss and preventable blindness in the working-age population [47]. This emphasis aligns with global epidemiologic projections showing that the number of adults aged 20–79 years living with DM is expected to rise from approximately 463 million in 2019 to roughly 700 million by 2045 [48]. The second cluster encompassed research on healthcare delivery systems, patient satisfaction, and cost-effectiveness, reflecting recognition that adherence to recommended DR screening remains suboptimal [49] and that telemedicine-based models may help address structural, logistical, and economic barriers that hamper patient participation in screening programs. The third cluster centered on machine learning-driven technologies, illustrating the growing efforts for integration of AI into teleophthalmology, particularly for retinal disorders. This rising interest is driven not only by the broader adoption of AI in medicine but also by the strong emphasis of ophthalmology on medical images, such as digital fundus photographs, which can be effectively handled and analyzed by deep learning algorithms [50].

Telemedicine can support ophthalmologic practice by enabling remote consultations, screening, supervision, and triage across a wide range of ocular diseases, demonstrating flexibility in meeting varying clinical needs. Notably, in a scoping review of 2019, DR represented the most common condition addressed within disease-specific teleophthalmology models. According to the same study, the predominant workflow of telecare delivery in ophthalmology involved on-site healthcare professionals capturing ocular images and transmitting them to remote specialized ophthalmologists for interpretation [51].

Teleophthalmology has shown potential to enhance DR screening, facilitate earlier access to eye care, and decrease healthcare costs [52]. Specifically, telemedicine using digital imaging has demonstrated an overall high diagnostic accuracy for DR [44]. Beyond diagnostic effectiveness, telemedicine has also been shown to improve adherence to DR screening guidelines. In a recent quasi-experimental study, implementation of a community-based telehealth screening program for DR in urban settings statistically significantly improved adherence to annual screening recommendations [53]. Similarly, a multicenter randomized controlled clinical trial demonstrated that offering patients with DM access to telemedicine can increase their likelihood of participating in DR screening [32]. Of note, telecare models for DR can also yield economic benefits. In a cost-effectiveness study by Curran et al. [54], telehealth screening for DR was not only as effective as conventional retinal examinations but also cost-saving over a one-year time horizon, reducing overall costs by approximately 28% compared to standard in-person visits.

DR is frequently overlooked largely because of its initially silent and asymptomatic course [55]. Meanwhile, early detection followed by timely intervention is crucial for preventing or delaying subsequent vision loss [56]. In line with this need, the American Academy of Ophthalmology’s Diabetic Retinopathy Preferred Practice Pattern recommends that individuals with type 1 DM undergo annual screening for DR beginning five years after disease onset, whereas those with type 2 DM should receive a prompt screening at the time of diagnosis followed by at least yearly examinations thereafter [57]. However, despite the documented benefits of early detection, only 50–60% of patients with DM in the United States adhere to these screening recommendations [58]. This documented underutilization of retinal screening, coupled with the projected global rise in the prevalence of DM in the upcoming years [48], underscores how teleophthalmology could play a pivotal role in improving the detection and management of DR. Evidence suggests that telemedicine-based screening programs may help bridge this gap by enabling more accessible, convenient, and affordable routes for patients with DM to obtain timely retinal evaluations [32,44,53,54].

We followed a rigorous methodology to conduct, what is to the best of our knowledge, the first bibliometric analysis examining telemedicine in the context of DR. However, some limitations should be acknowledged. First, our search strategy may not have been fully sensitive because it solely relied on the Web of Science Core Collection and was restricted to publications written in English, which may have resulted in incomplete coverage of the available global literature. Nevertheless, this potential omission of relevant studies is likely modest given that Web of Science is one of the largest and most comprehensive bibliographic databases worldwide and that the number of non-English articles in the field of interest was relatively small. This single-database approach was necessary to ensure consistency of bibliographic data and aligns with practices adopted in other high-impact bibliometric analyses [59,60]. Still, the exclusive use of Web of Science may have influenced our findings by underrepresenting pertinent studies published in newer journals not yet indexed in this database despite their potentially high scientific quality. Second, our literature search may not have achieved optimal specificity because, due to the large number of retrieved documents and the nature of bibliometric analysis, we did not screen the full texts of the included papers, creating the possibility that a number of articles not directly related to our topic may have been analyzed. Third, citation-based metrics are subject to temporal lag, meaning that some recently published but high-quality studies may have been underexplored in our analysis. Consequently, very recent advances and trends related to emerging technologies may have appeared less impactful than they actually are due to insufficient time to accumulate citations. Fourth, citation counts alone do not fully capture an article’s quality and scientific impact, as they are also affected by factors such as publication age, breadth of scope, and journal visibility, while self-citations may introduce bias.

Subsequent bibliometric work on telemedicine for DR should update the literature search beyond 2025 to capture the rapid developments currently underway and anticipated in the near future, particularly with the ongoing expansion of medical AI. Such research would also benefit from incorporating additional bibliographic databases, such as Scopus, which was not used in the present study. More broadly, research in this field could continue to move beyond feasibility and diagnostic performance toward real-world implementation studies that evaluate downstream clinical outcomes and equity across diverse healthcare settings. Such progress would be strengthened by a greater number of randomized controlled clinical trials, which remain relatively scarce in the current literature [32,61,62,63].

## 5. Conclusions

The findings of this bibliometric analysis indicate that the application of telemedicine in DR is an expanding research field, with contributions from multiple authors and institutions throughout the world. The concentration of pertinent studies within ophthalmology, endocrinology, and health informatics journals highlights the multidisciplinary nature of this field and the need for collaboration across clinical and technical domains in order to advance it. The thematic structure of the current literature reflects three major areas of research within the broader topic of telemedicine and DR, namely, population-based and public health-oriented work on epidemiology and screening, evaluations of teleophthalmology-based healthcare delivery and clinical service models, and applications of machine learning-driven systems. Collectively, these research directions illustrate a dynamic and evolving field that aims to support more accessible, efficient, and equitable identification and management of DR by leveraging ongoing technological advancements in telemedicine.

## Figures and Tables

**Table 1:** Top 10 countries with the largest number of publications in the field of telemedicine and diabetic retinopathy based on the corresponding author’s institutional affiliation.

Country	Articles (%)	MCPs (%)	Total Citations ^1^	Average Citations per Article
United States	207 (40.2)	37 (17.9)	7077	34.2
China	34 (6.6)	10 (29.4)	611	18.0
Australia	30 (5.8)	7 (23.3)	644	21.5
Canada	26 (5.0)	7 (26.9)	745	28.7
Spain	26 (5.0)	1 (3.8)	1619	62.3
India	25 (4.9)	5 (20.0)	410	16.4
Singapore	21 (4.1)	17 (81.0)	3849	183.3
United Kingdom	21 (4.1)	7 (33.3)	584	27.8
Italy	20 (3.9)	6 (30.0)	398	19.9
Brazil	15 (2.9)	4 (26.7)	197	13.1

^1^ Refers to the total number of citations each country’s articles within the bibliometric dataset have received across the entire Web of Science Core Collection. Abbreviation: MCPs, multiple-country publications.

**Table 2:** Top 10 journals with the highest number of publications in the field of telemedicine and diabetic retinopathy.

Journal	Category ^1^	Articles	Global Citations ^2^	Local Impact ^3^		
				*h*-Index ^4^	*g*-Index ^5^	*m*-Index ^6^
*Telemedicine and e-Health*	Health Informatics; Health Information Management; Medicine (miscellaneous)	66	1774	22	41	0.917
*Journal of Telemedicine and Telecare*	Health Informatics	20	641	14	20	0.538
*British Journal of Ophthalmology*	Cellular and Molecular Neuroscience; Ophthalmology; Sensory Systems	17	1405	11	17	0.647
*Canadian Journal of Ophthalmology*	Medicine (miscellaneous); Ophthalmology	15	440	11	15	0.44
*Jama Ophthalmology*	Ophthalmology	15	761	14	15	1.167
*Ophthalmology*	Ophthalmology	14	974	11	14	0.393
*Indian Journal of Ophthalmology*	Ophthalmology	13	217	9	13	0.643
*European Journal of Ophthalmology*	Medicine (miscellaneous); Ophthalmology	11	42	4	6	0.4
*Current Diabetes Reports*	Endocrinology, Diabetes, and Metabolism; Internal Medicine	10	385	10	10	0.588
*Ophthalmology and Therapy*	Ophthalmology	10	185	5	10	0.625

^1^ Based on the SCImago Journal Rank classification; ^2^ the total number of citations each journal’s articles within the bibliometric dataset have received across the entire Web of Science Core Collection; ^3^ calculated only from each journal’s articles within the bibliometric dataset; ^4^ the largest number *h* such that *h* articles have received at least *h* citations [23]; ^5^ the largest number *g* such that the top *g* articles have cumulatively received at least *g*^2^ citations [24]; ^6^ the *h*-index divided by the number of years since the publication of the first article [23].

**Table 3:** Top 10 authors with the largest number of publications in the field of telemedicine and diabetic retinopathy.

Author	Articles	Local Citations ^1^	Global Citations ^2^	Local Impact ^3^		
				*h*-Index ^4^	*g*-Index ^5^	*m*-Index ^6^
Cavallerano, JD	25	334	1056	18	25	0.818
Silva, PS	24	147	673	14	24	0.824
Aiello, LP	18	188	718	14	18	0.636
Ting, DSW	17	191	3511	13	17	0.867
Aiello, LM	13	206	603	12	13	0.545
Tennant, MTS	12	206	474	11	12	0.440
Bursell, SE	11	188	552	9	11	0.409
Horton, MB	10	149	537	10	10	0.455
Wong, TY	10	176	3276	9	10	0.900
Sun, JK	9	90	351	7	9	0.500

^1^ The number of citations each author’s articles within the bibliometric dataset have received from other articles in the same dataset; ^2^ the total number of citations each author’s articles within the bibliometric dataset have received across the entire Web of Science Core Collection; ^3^ calculated only from each author’s articles within the bibliometric dataset; ^4^ the largest number *h* such that *h* articles have received at least *h* citations [23]; ^5^ the largest number *g* such that the top *g* articles have cumulatively received at least *g*^2^ citations [24]; ^6^ the *h*-index divided by the number of years since the publication of the first article [23].

**Table 4:** Top 10 original and top 10 review articles with the highest total citation count in the field of telemedicine and diabetic retinopathy.

Title	Authors, Year	Main Affiliation ^1^	Journal	Global Citations ^2^	Local Citations ^3^
Original Articles					
Development and Validation of a Deep Learning System for Diabetic Retinopathy and Related Eye Diseases Using Retinal Images From Multiethnic Populations With Diabetes [25]	Ting et al., 2017	Singapore National Eye Center	*JAMA*	1500	53
Improved Automated Detection of Diabetic Retinopathy on a Publicly Available Dataset Through Integration of Deep Learning [26]	Abràmoff et al., 2016	University of Iowa	*Investigative Ophthalmology & Visual Science*	717	36
A New Supervised Method for Blood Vessel Segmentation in Retinal Images by Using Gray-Level and Moment Invariants-Based Features [27]	Marín et al., 2011	University of Huelva	*IEEE Transactions on Medical Imaging*	689	0
Detecting the Optic Disc Boundary in Digital Fundus Images Using Morphological, Edge Detection, and Feature Extraction Techniques [30]	Aquino et al., 2010	University of Huelva	*IEEE Transactions on Medical Imaging*	342	1
American Association of Clinical Endocrinology Clinical Practice Guideline: Developing a Diabetes Mellitus Comprehensive Care Plan-2022 Update [34]	Blonde et al., 2022	University of South Carolina	*Endocrine Practice*	287	0
Artificial intelligence for teleophthalmology-based diabetic retinopathy screening in a national programme: an economic analysis modelling study [28]	Xie et al., 2020	National University of Singapore	*The Lancet Digital Health*	183	22
Cost-effectiveness of a National Telemedicine Diabetic Retinopathy Screening Program in Singapore [31]	Nguyen et al., 2016	Singapore National Eye Center	*Ophthalmology*	155	48
Long-term Comparative Effectiveness of Telemedicine in Providing Diabetic Retinopathy Screening Examinations: A Randomized Clinical Trial [32]	Mansberger et al., 2015	Legacy Health	*JAMA Ophthalmology*	146	69
The cost-utility of telemedicine to screen for diabetic retinopathy in India [33]	Rachapelle et al., 2013	London School of Hygiene & Tropical Medicine	*Ophthalmology*	127	55
Prospective evaluation of an artificial intelligence-enabled algorithm for automated diabetic retinopathy screening of 30 000 patients [29]	Heydon et al., 2021	University of London	*British Journal of Ophthalmology*	110	12
**Review Articles**					
Artificial intelligence and deep learning in ophthalmology [35]	Ting et al., 2019	Singapore National Eye Center	*British Journal of Ophthalmology*	866	19
Digital technology, tele-medicine and artificial intelligence in ophthalmology: A global perspective [36]	Li et al., 2021	Singapore National Eye Center	*Progress in Retinal and Eye Research*	368	17
Cost-utility and cost-effectiveness studies of telemedicine, electronic, and mobile health systems in the literature: a systematic review [39]	de la Torre-Díez et al., 2015	University of Valladolid	*Telemedicine and e-Health*	322	5
Screening and prevention of diabetic blindness [40]	Stefánsson et al., 2000	Malmö University	*Acta Ophthalmologica Scandinavica*	221	9
Using Digital Health Technology to Better Generate Evidence and Deliver Evidence-Based Care [41]	Sharma et al., 2018	Stanford University	*Journal of the American College of Cardiology*	208	0
Fundus Photography in the 21st Century--A Review of Recent Technological Advances and Their Implications for Worldwide Healthcare [42]	Panwar et al., 2016	Tan Tock Seng Hospital	*Telemedicine and e-Health*	190	14
The Current State of Teleophthalmology in the United States [43]	Rathi et al., 2017	New York University	*Ophthalmology*	175	33
Artificial Intelligence Screening for Diabetic Retinopathy: the Real-World Emerging Application [37]	Bellemo et al., 2019	Singapore National Eye Center	*Current Diabetes Reports*	117	10
Telemedicine for detecting diabetic retinopathy: a systematic review and meta-analysis [44]	Shi et al., 2015	Nantong University	*British Journal of Ophthalmology*	117	37
The current state of artificial intelligence in ophthalmology [38]	Kapoor et al., 2019	Columbia University	*Survey of Ophthalmology*	110	5

^1^ Refers to the institutional affiliation listed in the corresponding author’s address; ^2^ the total number of citations an article has received across the entire Web of Science Core Collection; ^3^ the number of citations an article has received from other articles in the bibliometric dataset.

## Data Availability

No new data were created or analyzed in this study.

## Abbreviations

The following abbreviations are used in this manuscript:

AI	artificial intelligence
COVID-19	coronavirus disease 2019
DM	diabetes mellitus
DR	diabetic retinopathy
